# Membrane Nanofiber-Supported Cobalt–Nickel Nanoparticles as an Effective and Durable Catalyst for H_2_ Evolution via Sodium Borohydride Hydrolysis

**DOI:** 10.3390/polym15040814

**Published:** 2023-02-06

**Authors:** Nasser Zouli, Ibrahim M. Maafa, Ahmed Abutaleb, Ayman Yousef, M. M. El-Halwany

**Affiliations:** 1 Department of Chemical Engineering, College of Engineering, Jazan University, Jazan 45142, Saudi Arabia; 2 Department of Mathematics and Physics Engineering, College of Engineering in Matteria, Helwan University, Cairo 11718, Egypt; 3 Department of Mathematics and Physics Engineering, College of Engineering, Mansoura University, El-Mansoura 35516, Egypt

**Keywords:** electrospinning, bimetallic NiCo, PVDF-HFB nanofibers, hydrogen, sodium borohydride

## Abstract

The successful support of bimetallic NiCo alloy nanoparticles (NPs) on poly(vinylidene fluoride-co-hexafluoropropylene) nanofibers (PVDF-HFP NFs) was achieved through electrospinning (ES) and in situ reduction. The synthesis and physicochemical characterization of Ni-Co@PVDF-HFP NFs with a range of bimetallic compositions (Ni_1−x_Co_x_, x = 0, 0.1, 0.3, 0.5, 0.7, 0.9, and 1) supported on PVDF-HFP NFs was undertaken. In comparison to their counterparts (Ni-PVDF-HFB and Co-PVDF-HFB), the bimetallic hybrid NF membranes demonstrated a significantly increased volume of H_2_ generation from sodium borohydride (SBH). The high performance of bimetallic catalysts can be attributed mostly to the synergistic impact of Ni and Co. Among all fabricated catalysts, Ni_0.3_Co_0.7_@PVDF-HFP produced the highest H_2_ production in a short time. The maximum generated H_2_volume was 118 mL in 11.5, 9, 6, and 4.5 min at 298, 308, 318, and 328 K, respectively. Kinetic analyses showed that the hydrolysis process proceeded as a quasi-first-order reaction with respect to the amount of catalyst and as a zero-order reaction with respect to the concentration of SBH. Thermodynamics studies were also undertaken and the parameters were calculated as *E_a_*, Δ*S*, and Δ*H* = 30.17 kJ/mol, 0.065 kJ/mol, and 27.57 kJ/mol K, respectively. The introduced NFs can be easily separated and reused, which facilitates their industrialization and commercialization applications in hydrogen storage systems.

## 1. Introduction

Sodium borohydride (NaBH_4_, SBH) is commonly recognized as a potential material for hydrogen storage owing to its ability to produce high-purity H_2_ gas (>99%) at a very fast rate at ambient temperature. By using appropriate catalysts, as stated in Equation (1) [1,2,3], 4 moles of H_2_ are generated, with 50% of the hydrogen coming from water.

(1) $${\mathrm{NaBH}}_{4}+2H_{2}O\overset{\mathrm{catalyst}}{\to }4H_{2}+{\mathrm{NaBO}}_{2}$$

Although Pt (USD 31,783/kg Pt) and Ru (USD 8504/kg Ru) are the best metallic catalysts for the dehydrogenation of SBH due to their outstanding catalytic activity [4,5,6], their high cost may prohibit their usage in many H_2_-generation applications. For this reason, there has been a significant amount of interest in finding substitutes for noble-metal catalysts, such as cobalt (Co; USD 32/kg Co) and nickel (Ni; USD 17/kg Ni) [6,7,8]. Nowadays, they are the most extensively utilized metals in H_2_ production from SBH due to their low-cost and abundance compared to noble metals [4,9,10,11,12]. Kaufman and Sen found that heterogeneous catalysts such as Co and Ni may alter the behavior of BH_4_^−^hydrolysis by transferring electrons to water molecules, allowing for the production of H_2_ gas [13]. There are a few drawbacks to using nanoparticles (NPs) directly as a catalyst for the hydrolysis of NaBH_4_. NPs with high surface energies agglomerate significantly during the reaction, reducing their catalytic activity and making them unusable for reuse. A further major practical challenge is removing the nano-powder from the reaction medium [14]. In order to prevent nanoparticles from clustering together, it is recommended to load them onto a suitable support [15]. The dispersion and stability of active metal nanoparticles (MNPs) are two critical elements in the design and production of highly effective catalysts for SBH hydrolysis. Various materials have been utilized as supports for NPS (e.g., rGO, Al_2_O_3_, TiO_2_, Cu sheets, Ni foam, carbon cloth, etc.) and their ability to hydrolyze NaBH_4_has been tested [16,17,18,19,20,21]. The catalyst performance and longevity are profoundly affected by the supporting materials [22]. As is well known, polymer substrates can readily be separated from reactants while retaining their adaptable design structures. As a consequence, a wide range of Ni and Co materials supported on polymer matrices with distinct shapes and structures have been prepared [23,24,25,26,27,28,29,30,31,32,33,34,35,36]. They overcame the aforementioned challenges while proving to be very useful in the production of H_2_ from NaBH_4_. As is known, the catalytic activity of catalysts varies greatly depending on both their preparation method and their shape. It has been claimed that various polymeric hydrogel networks have been used as a supporter for Ni and Co nanoparticles and as soft reactors in the catalysis of H_2_ generation [37,38]. A polymer nanofiber membrane (PNFM) has been suggested as a suitable support material for NPs in a wide range of chemical processes. PNFM may be reusable and recycled more effectively than other support materials. PNMs produced through the electrospinning method have recently been recognized as a novel category of porous materials due to their high porosity, large surface area, and chemical tenability [39,40]. Heterogeneous catalysts might be made possible through the deposition of metal NPs onto PNM. The nanoporous architectures of PNMs are thought to decrease the probability of aggregation of the active components, leading to long-term stability. Composite NFs prepared by anchored cobalt(II)chloride on polyacrylonitrile membrane NFs demonstrated superior catalytic activity in hydrogen generation from SBH [41]. Kim and his group have reported supported Y-zeolite/CoCl_2_[22] and nickel NPs [42] on PVDF membranes. The synthesized hybrid NFs demonstrated excellent catalytic activity towards hydrogen generation from SBH. Recently, our group prepared Ni NPs supported on a PVDF-co-HFP membrane [43]. The prepared NFs showed superior catalytic performance and reusability in hydrogen generation from SBH. As a host matrix for making hybrid composites, PVDF-HFP is well recognized [44]. It absorbs electrolyte solutions with a high affinity, has great chemical and electrochemical stability, and is an easily recyclable polymer [45].

In this study, bimetallic NiCo supported well on a PVDF-HFP matrix was fabricated using the electrospinning technique and in situ reduction approach. ES nanofibers consist of CoAc, NiAc, and PVDF-co-HFP and are dried and reduced in situ with SBH in a methanol solution to produce NiCo NPs supported on a PVDF-co-HFP membrane. Based on the results of the physicochemical characterizations undertaken, it was determined that the reduction process results in the formation of Ni-Co supported on PVDF-co-HFP NFs. Bimetallic Ni-Co deposition improves PVDF-co-HFP by lowering its crystallinity and increasing solution absorption, which may improve SBH–catalyst surface contact [44]. To our knowledge, this is the first time Ni-Co NPs@ PVDF-co-HFP membrane NFs have been synthesized for use in hydrogen evolution via dehydrogenation of SBH as a highly convenient and easily recyclable catalyst. According to the findings, the rate of dehydrogenation of SBH is mostly determined by the amount of catalyst, the temperature of the reaction, and the initial concentration of SBH in the reaction. To that end, this research looked at the potential of Ni-Co NPs supported on PVDF-co-HFP NFs as inexpensive catalysts for H_2_ production from SBH.

## 2. Experimental Section

### 2.1. Materials

Sodium borohydride (NaBH_4_, SBH), cobalt (II) acetate tetrahydrate (CoAc), nickel (II) acetate tetrahydrate (NiAc), and PVDF-co-HFP [(MW) = 65,000 g/mol] were used. N,N-dimethylformamide (DMF) and acetone were used as a solvent. All chemicals were purchased from Aldrich Co., St. Louis, MO, USA

### 2.2. Synthesis of Bimetallic NiCo Supported on PVDF-co-HFP

By mixing 1.5 g of PVDF-HFP in a blend of 4:1 DMF and acetone, a solution of PVDF-HFP with 15 wt.% was obtained. The prepared solution was electrospun using a laboratory-scale electrospinner (Nano Fiber Lab, Foshan, China) to produce membrane NF mats. The electrospinning machine is composed of a plastic capillary syringe (Nahdi Pharmacy, Jazan, Saudi Arabia) loaded with the as-prepared solution. A conductive copper wire was passed through the solution and connected to a high-voltage power supply which served as a positive electrode, while an iron rotating drum covered with aluminum foil served as the negative electrode. A high voltage (20 kV) was applied between the syringe and drum. The distance between drum and syringe was kept at 18 cm throughout the experiment. After collecting the electrospun NF mats, they were dried in a vacuum overnight at 50 °C to remove the remaining solvents. To prepare the composite NF membranes, stock solutions from different compositions of Ni_x_Co_1−x_(x = 0, 0.1, 0.3, 0.5, 0.7, 0.9, and 1) were prepared by dissolving precursors in DMF. In the separated glass bottles, solutions with different compositions were added to the as-prepared 15% PVDF-co-HFP solution to load the metal ions into PVDF-co-HFP matrices. The concentrations of precursors were kept at 40% based PVDF-co-HFP for all formulations. The solutions were stirred for 5 h to obtain well-mixed sol–gels. The same electrospun and drying conditions were used for the preparation of the NF membranes with different formulations.

### 2.3. Preparation of Ni_x_Co_1−x_ Supported on PVDF-co-HFP Membrane

Part of the PVDF-co-HFP membrane was soaked in a glass beaker containing 100 mL of methanol solution. The aforementioned solution was agitated at a constant rate of 1000 rpm, then SBH was added slowly in molar ratios 1:5 (SBH: metal ions). These reactions were performed in an ice bath to prevent the solution from undergoing an extreme reaction. A noticeable color change was observed in the mats as soon as SBH attached to the mats. Mats that were originally green (indicating a high concentration of NiAc) or purple (indicating a high concentration of CoAc) were shown to undergo a reduction in metallic Ni, Co, or NiCo on the surface of PVDF-co-HFP when their color shifted to black. The mats were left immersed in the solution until the gas bubbles had disappeared. Mats were washed multiple times in DI water and ethanol to remove free metal ions from PVDF-co-HFP. Finally, the formed membrane was dried at 50 °C overnight. After the hybrid membranes were produced, they were used as a catalyst in the hydrolysis of SBH.

### 2.4. Characterization

The physicochemical characterizations of the synthesized membranes were performed using our previously reported techniques [43].

### 2.5. H_2_ Evolution from SBH Using Synthesized Catalytic Membrane NFs

A 75 mL two-neck flask containing 100 mg of catalytic membranes was submerged in a water bath to regulate the temperature. The flask was tightly sealed with a stopper. Then, 50 mL of 1 mmol SBH solution was injected into the reaction flask using the syringe while the hotplate and stirrer (Sterlitech Co., Auburn, AL, USA) were used to rapidly magnetically stir the mixture at a speed of 1000 rpm. A thermocouple (Generic Co., Shanghai, China) was utilized in order to adjust the temperature of the reaction. The evolved H_2_ was collected in a burette via the water displacement technique, which entails passing the gas through a plastic tube and then flipping the burette upside down. The volume of evolved H_2_ was easily determined by measuring the height of the water in the burettes and then observing how much that level changed over the course of different time. Finally, a graph was constructed by plotting the volume of H_2_ gas that was produced against the time that had elapsed. All prepared catalysts were tested under the same conditions. Various quantities of the best catalyst (100, 150, 200, and 250 mg), concentrations of SBH (1, 2, 3, and 4 mmol), and temperatures (298, 308, 318, and 328 K) were studied for determining the hydrolysis kinetics. In addition, the recycling performance of the recently developed membranes was assessed. In order to determine the robustness of the catalyst, this procedure was carried out using the same catalytic membranes throughout all cycles. During each cycle, 1 mmol of SBH was added, 100 mg of catalyst, and the temperature was fixed at 25 °C throughout.

## 3. Results and Discussion

### 3.1. Characterization of Hybrid Nanofiber Mats

**Figure 1:**
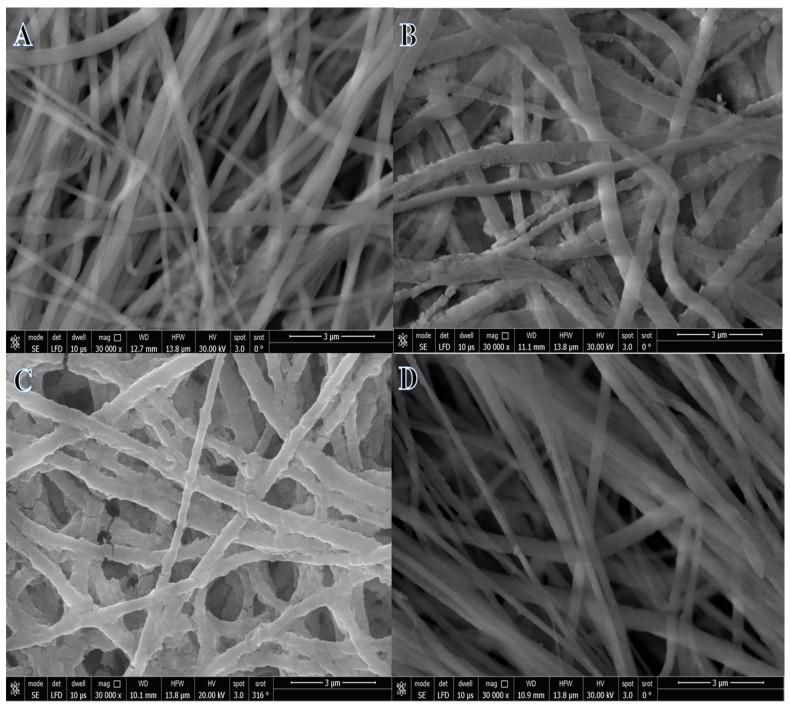
SEM images of PVDF*-*co*-*HFP (**A**), Ni@PVDF*-*co*-*HFP (**B**), Ni_0.3_Co_0.7_@PVDF*-*co*-*HFP (**C**), and Co@PVDF*-*co*-*HFP (**D**).

**Figure 2:**
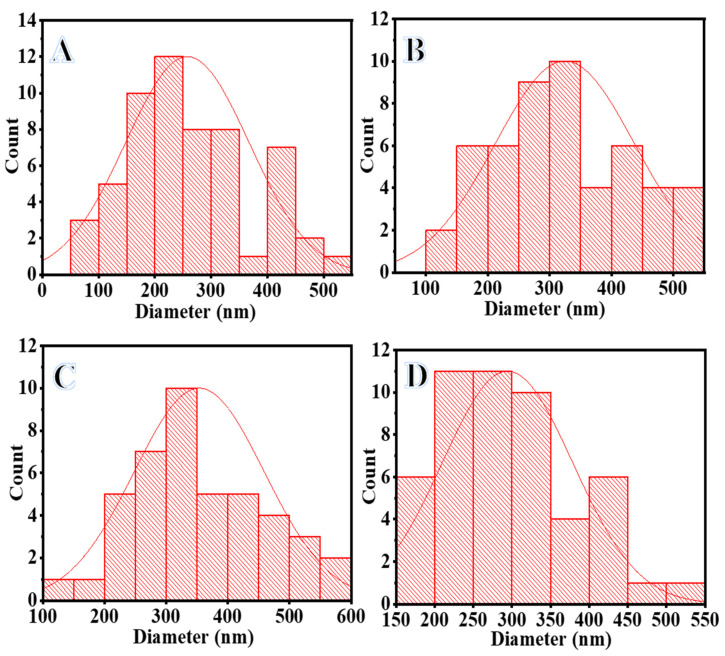
Size distribution of PVDF*-*co*-*HFP (**A**), Ni@PVDF*-*co*-*HFP (**B**), Ni_0.3_Co_0.7_@PVDF*-*co*-*HFP (**C**), and Co@PVDF*-*co*-*HFP (**D**).

Generally, the electrospinning process is widely used in producing organic, inorganic, and organic/inorganic NFs. It is one of the methods that can be used to produce a polymeric nanofibrous membrane. This method can potentially have several advantageous properties, including increased interconnectivity, flexibility, perfect porosity, and exceptional surface-to-volume ratios [46,47]. In the case of dissolved metal precursors mixed with polymer solution, they hydrolyzed and poly-condensate to form the gel network. During the electrospinning process, the gel network produced completely miscible NF mats. Due to the polycondensation properties of the formed NF mats maintaining a nanofibrous morphology, bead-free, continuous, and smooth NF mats with a large surface-area-to-volume ratio were usually obtained. Accordingly, an excellent nanofibrous structure can be seen in the SEM image of dried ES PVDF-co-HFP NF mats (Figure 1A). PVDF-co-HFP is considered a suitable polymer for producing membrane NFs due to distinct features such as its semi-crystalline nature, excellent thermal stability, hydrophobicity, and piezo- and pyroelectric characteristics. During the electrospinning process, the solvent tended to evaporate between the positive electrode and the negative electrode. Thus, nano-pores could be formed on the surface of electrospun NF mats, which could be ideal nucleation sites for metallic NPs. Figure 1B–D shows SEM images of electrospun Ni@PVDF-co-HFP, Co@PVDF-co-HFP, and Ni_0.3_Co_0.7_@PVDF-co-HFP membrane NFs, respectively. As seen in the figures, nanofibrous, rough, and bead-free structures are formed. Moreover, the addition of metals resulted in an increase in the NFs’ diameter as follows: 257.7 nm for PVDF-co-HFP (Figure 2A), 324.3 nm for Ni@ PVDF-co-HFP (Figure 2B), 353.5 nm for Ni_0.3_Co_0.7_@ PVDF-co-HFP (Figure 2C), and 299 nm for Co@ PVDF-co-HFP (Figure 2D). This increase in NF diameter may be attributed to the deposition of metallic NPs around the polymeric NFs.

As a strong reducing agent in methanol medium, SBH is used to reduce Ni and Co ions during in situ reduction. As soon as SBH is introduced, membranes change from their original hue to a black color. It is suggested that a thin layer of black Ni and Co dots, corresponding to the shell “Ni and Co nanoparticles” (the core) “polymeric NF” configurations, covers the whole surface of the PVDF-co-HFP membranes.

Since sodium tetramethoxy borate is insoluble in methanol, it is precipitated out during the reducing process alongside the metallic nanoparticles (NPs), which may help to keep the NPs apart and avoid their agglomeration. Methanol, as opposed to water, can be used for the dehydrogenation of NaBH_4_, which results in a few advantages. Sodium tetramethoxy borate is produced as a byproduct of methanolysis, and it does not readily form polyborate complexes. Furthermore, it is capable of preventing catalyst poisoning by avoiding the precipitation of NaB(OH)_4_ [48,49]. Sodium tetramethoxy borate reacts with water to produce sodium borate and methanol, both of which are carried away by the washing process’s excess water. As a result, the membrane’s surface will be riddled with nano-pores. This would allow the SBH molecules to be trapped in the pores with the least level of resistance to diffusion feasible, simplifying the process of H_2_ evolution. Fine Co NPs@carbon nanomaterials were synthesized by Hongming et al. [50] using hydrothermal and reduction processes, and they have shown to be a convenient catalyst for H_2_ evolution from the dehydrogenation of SBH. When comparing the reduction of metal ions in water and ethyl alcohol solution media, they observed that the former was better due to the poor solubility of sodium metaborate on ethanol. In water, some sodium metaborate flowed out with the Co NPs. This assisted in dispersing and preventing the clumping of Co nanoparticles. Finally, DI water was utilized to flush out the sodium metaborate.

**Figure 3:**
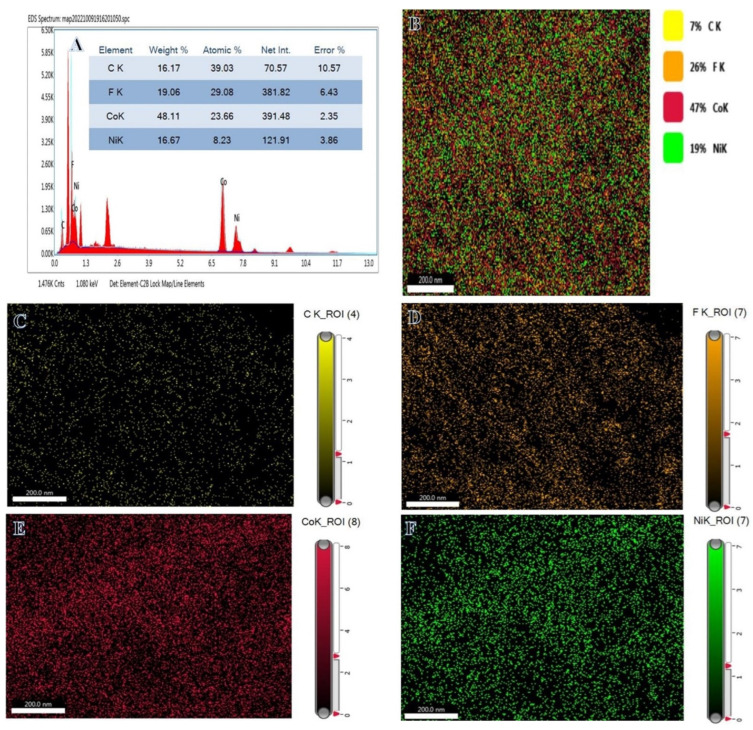
EDX chart of Ni_0.3_Co_0.7_@PVDF*-*co*-*HFP NFs membrane (**A**) and mapping showing the spread of elements (**B**), carbon (**C**), fluorine (**D**), cobalt (**E**), and nickel (**F**) in the Ni_0.3_Co_0.7_@PVDF*-*co*-*HFP NFs.

Figure 3A displays the EDX spectrum of Ni_0.3_Co_0.7_@PVDF-co-HFP membrane NFs. As shown in Figure 3A, the product is made up of carbon, fluorine, nickel, and cobalt in its chemical makeup. The wt.% of elements is shown in the table inset in Figure 3A. It is indicated that the composition is extremely close to that of the starting precursors. The elemental mapping image of the Ni_0.3_Co_0.7_@ PVDF-co-HFP membrane NFs is shown in Figure 3B–F. Ni and Co NPs could be observed widely dispersed throughout the membrane NFs.

**Figure 4:**
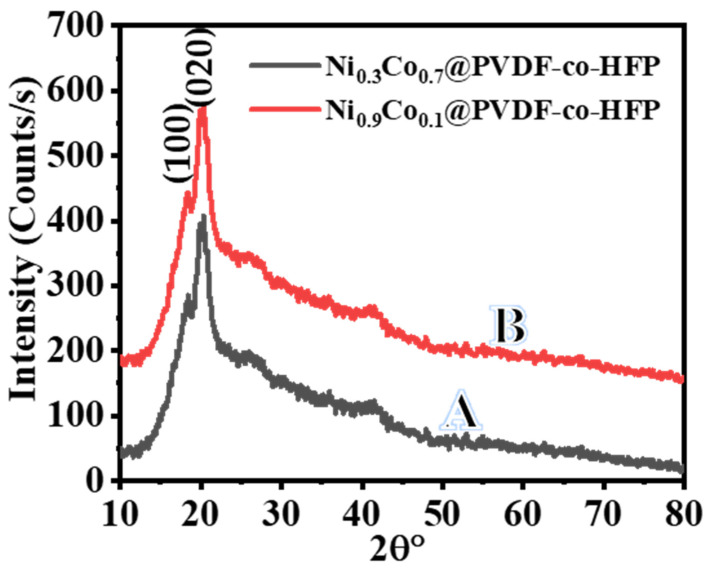
XRD patterns of Ni_0.3_Co_0.7_@PVDF*-*co*-*HFP (A) and Ni_0.9_Co_0.1_@PVDF*-*co*-*HFP (B) NF membranes.

XRD analysis of Ni_0.3_Co_0.7_@PVDF-HFP and Ni_0.3_Co_0.7_@PVDF-HFP membranes is shown in Figure 4A,B, respectively. It is obvious that two primary diffraction peaks exist at a 2θ of 18.4° and 20.3°, which correspond to the (100) and (020)crystal indices, respectively [51]. In contrast, no XRD peaks related to Ni or Co NPs existed in the sample either because Ni and Co NPs are ultrafine, or the formed Ni-Co was non-crystalline [52,53,54].

**Figure 5:**
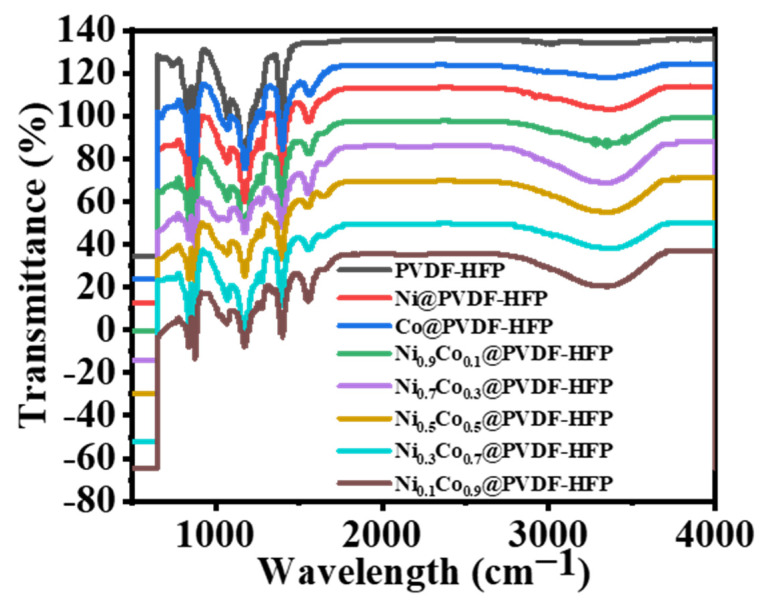
FTIR analysis of PVDF*-co-*HFP, Ni@PVDF*-*co*-*HFP, Co@PVDF*-*co*-*HFB, and Ni_0.3_Co_0.7_@PVDF*-*co*-*HFP NF membranes.

Figure 5 gives the FTIR spectra of all as-prepared membranes. Charts showing the polymeric membranes showed bands of vibrational modes that were similar. The α and β phases of PVDF-HFP were verified by peaks at 749 and 837 cm^−1^ [55]. Vibrational bands of vinylidene units were identified as the CF wagging at 672 cm^−1^ and the CH_2_ wagging at 872 cm^−1^ in the non-crystalline phase of the PVDF-co-HFP matrix. There were further bands at 1400, 1175, and 1071 cm^−1^ that demonstrated the deformed vibrations, CF_2_ stretching, and symmetric CF stretching [56]. When metal precursor salts were used into the production of the polymeric films, new peaks developed. This was further supported by the fact that the stretching vibration maxima of Co-O and Ni-O species were seen around 1567 cm^−1^ [57]. Other bands can be seen at 3360 cm^−1^, characteristic of the OH or B-OH stretching mode, suggesting that some of the NaBH_4_ was converted into a borate (amorphous and unable to be analyzed by XRD) during the reduction process in methanol media, and at 1644 cm^−1^, characteristic of the OH deformation mode [48,58,59].

### 3.2. Catalytic Hydrolysis of Ammonia Borane

#### 3.2.1. Effect of Monometallic (Ni and Co) Nanoparticles @PVDF-co-HFP and Bimetallic Nanoparticle Composition @PVDF-co-HFP in the Hydrolysis Reaction

**Figure 6:**
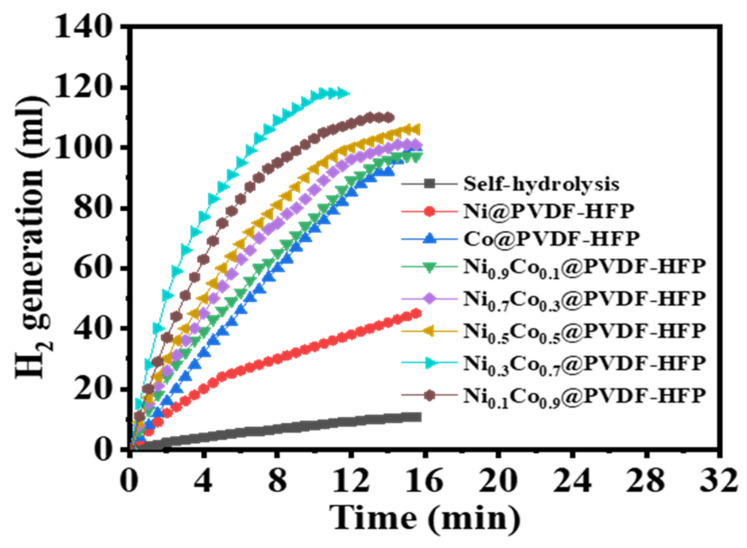
Effect of Ni_x_Co_1−x_@PVDF-co-HFP NFs on the dehydrogenation of SBH. [Quantity of catalyst = 100 mg, [SBH] = 1 mmol, and T = 298 K].

It is well known that the same reaction can be catalyzed at varying rates using different monometallic nanoparticles or different bimetallic compositions. In this investigation, seven different prepared supported catalysts were utilized in order to evaluate and compare their catalytic activities in the dehydrogenation of SBH under identical circumstances. The operating conditions of the experiments consist of 100 mg of each catalyst, 1 mmol of SBH, 25 °C, and 1000 rpm. The volume of H_2_ production from SBH utilizing monometallic@PVDF-co-HFP and bimetallic@PVDF-co-HFP NF membrane catalysts as a function of the reaction time is shown in Figure 6. By comparing the prepared catalysts, it can be observed that bimetallic@PVDF-HFP NF membrane catalysts exhibit a higher volume of hydrogen compared to their counterparts. Even while bimetal catalysts have been shown to have greater catalytic activity over comparable single metals, similar results have been observed with other metal catalysts [8,17,37,60,61,62]. Figure 6 displays the rapid growth of the response rate at the outset of the study period. Here, it appears that the surface of the metal catalyst is where the catalytic reaction occurs [63]. Metal-free PVDF-co-HFP membranes were employed to evolve meager amounts of H_2_. These results strongly suggest that a synergistic connection between Co and Ni plays a crucial role in the dehydrogenation of SBH. Both electrical aspects, which were significant when the metal atoms have varied electronegativity [64], and geometric effects contribute to the synergistic action observed in alloy NPs. Since Co and Ni have electronegativities of 1.88 and 1.91, respectively, it is probable that the geometric and electronic impacts contribute most to the observed synergetic effects in catalysis. By forming CoNi alloy NPs, Ni can partially replace Co in the Co structure without altering the A1-type lattice structure of the Co crystal system, resulting in potent synergistic interactions between the two metals. Furthermore, the host polymer (PVDF-co-HFP) might provide a good dispersion of metal NPs on its surface, which produces a large number of active sites and a high absorptive capacity of SBH. It is well known that the electrospun NFs have an excellent nano-porous architecture that promotes interaction between reactants and catalytic metals and facilitates the evolution of H_2_. This is because the nanofibrous structure provides a large surface area and active sites for H_2_ generation through SBH hydrolysis. The bimetallic catalyst Ni_0.3_Co_0.7_ had the highest catalytic activity, releasing 118 mL H_2_ in 11.5 min compared to the other four bimetal catalysts Ni_0.9_Co_0.1_(110 mL H_2_ in 15 min), Ni_0.5_Co_0.5_(106 mL H_2_ in 16 min), Ni_0.3_Co_0.7_(101 mL H_2_ in 16 min), and Ni_0.1_Co_0.9_(97 mL H_2_ in 16 min). The order of H_2_ released in as follows: Ni_0.3_Co_0.7_ > Ni_0.1_Co_0.9_ > Ni_0.5_Co_0.5_ > Ni_0.3_Co_0.7_ > Ni_0.9_Co_0.1_. It was discovered that adjusting the Co/Ni ratio affected the catalytic performance of NiCo. It was found that the catalytic performance of all five bimetal catalysts improves with increasing Co wt.%. The Co is, thus, more likely to catalyze the hydrolysis of SBH. This could be attributed to the fact that Co offers a higher catalytic performance than that of Ni in H_2_ production from hydrogen storage materials [61]. The increase in the concentration of Co (Ni_0.1_Co_0.9_@PVDF-co-HFP) lead to a decrease in the catalytic performance, which may be due to the agglomeration of Co and Ni NPs on the surface of PVDF-co-HFP that decrease the NPs surface area and catalyst activity. According to these findings, Ni may be able to promote the transfer of more electric charge to Co, thereby increasing the number of active sites on the surface of the Co atoms, which promotes the reaction kinetics. Furthermore, the combination of nickel and cobalt in an alloy improves the formation of an electrophilic or Lewis acid site, which facilitates the absorption and activation of reactants, ultimately leading to an increase in the reaction rate of the catalyst as a whole. BH_4_^−^ ions are chemically bound to the Co atoms through chemisorption [65]. H^−^ is moved from the BH_4_^−^atom to the Co atom. The H atom picks up an electron from Co, resulting in the formation of the H^−^ hydridic form; however, the BH_3_^−^ species maintains its attachment to the cobalt atom. When this H^−^ combines with a water molecule, the resulting reaction produces H_2_ and OH^−^. OH^−^ and BH_3_^−^ undergo a chemical reaction that results in the formation of the BH_3_(OH)^−^ ion. Additional H^−^ is moved from the BH_3_(OH)^−^ ion to an adjacent unoccupied Co atom. According to Patel et al. [66], however, during the final step, the reaction between OH^−^ and BH_3_^−^ ions is not promoted by the catalyst, which may produce a slower reaction rate. Ni may function as Lewis acid sites in the present catalyst. These sites are easily available for the uptake of Lewis bases such as OH ions. Therefore, the electron density on the active metal atoms will increase, which will favor the reaction kinetics.

#### 3.2.2. Effect of the Catalyst Amount in the Hydrolysis Reaction

**Figure 7:**
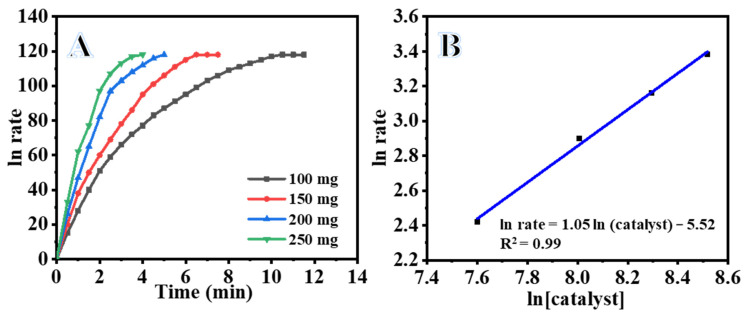
Effect of Ni_0.3_Co_0.7_@PVDF*-*co*-*HFP NFs on the dehydrogenation of SBH (**A**) and the log of the H_2_ generation rate vs. log of catalyst quantity (**B**). ([SBH] = 1 mmol and T = 298 K).

Figure 7A shows plots of the volume H_2_ versus the hydrolysis reaction time of SBH in the presence of different amounts of Ni_0.3_Co_0.7_@PVDF-co-HFPNF membrane catalysts in the range of 100 mg to 250 mg under the same operating conditions. The volume H_2_ increased and gradually decreased with the hydrolysis reaction time as the amount of Ni_0.3_Co_0.7_@PVDF-co-HFPNF membrane catalyst increased. The H_2_ generation rates were found to be 11.3, 18.2, 23.6, and 29.5 mL/min for the catalyst containing 100, 150, 200, and 250 mg of Ni_0.3_Co_0.7_@PVDF-co-HFPNF membrane catalyst. Plotted logarithmic H_2_ generation rates were obtained from the linear portion of each plot for each amount in Figure 7B versus logarithmic catalyst amounts; the slope of the straight fitting line is 1.04, which demonstrates that the hydrolysis reaction followed a quasi-first-order reaction with respect to the amount of the catalyst [37]. Based on these findings, an amount of 100 mg of Ni_0.3_Co_0.7_@PVDF-co-HFPNF membrane catalysts was selected for use in all the kinetic studies involving SBH hydrolysis reactions.

#### 3.2.3. Effect of Initial SBH Concentration in the Hydrolysis Reaction

**Figure 8:**
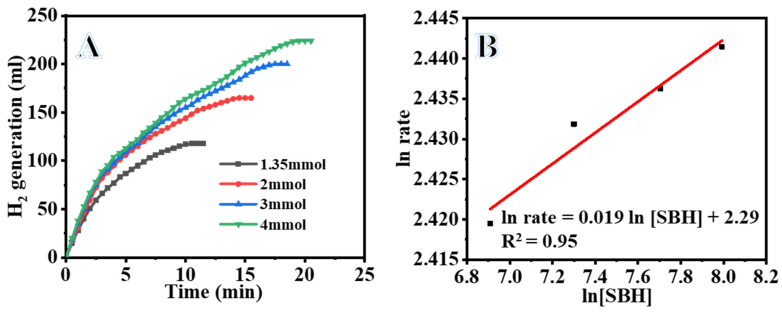
Effect of [SBH] on H_2_ evolution (**A**) and log of the H_2_ generation vs. log [SBH] (**B**). [Quantity of catalyst = 100 mg of Ni_0.3_Co_0.7_@PVDF-HFP NFs membrane and T = 298 K].

Figure 8A shows the results of H_2_ generation investigated at four different SBH concentrations (1, 2, 3, and 4 mmol) at 25 °C using 100 mg of Ni_0.3_Co_0.7_@PVDF-co-HFP NF membranes. Obviously, the evolved H_2_ linearly increases as SBH concentration increases from 1 mmol to 4 mmol in the reactor. This illustrates that the availability of SBH is irrelevant to H_2_ evolution, in as much as the rate of hydrolysis is the same regardless of the initial dosage of SBH. The amount of hydrogen gas produced was directly proportional to the amount of SBH used. In a reaction with 1 mmol NaBH_4_, 118 mL H_2_ was produced in 11.5 min; in a reaction with 4 mmol SBH, 224 mL H_2_ was evolved in 20 min. The time required to evolve a given volume of H_2_ increased as the concentration of SBH in the starting mixture increased. The results achieved here were consistent with those found in earlier studies [38,61] involving the use of a bimetallic CoNi catalyst for hydrogen evolution from SBH. The plotted logarithmic hydrogen evolve rates were obtained from the linear portion of each plot for each concentration in Figure 8B versus logarithmic SBH concentration. The slope of the straight fitting line is 0.019, indicating that the reaction is zero order. Zhu et al. [67] carried out an investigation on the hydrolysis of SBH using a supported cobalt catalyst on carbon. Their findings suggested that the generation rate increased linearly as the SBH content increased from 1 to 10%.

As is known, the presence of NaOH increased the efficiency of metal NPs as catalysts for the hydrolysis of NaBH_4_. This phenomenon has been linked to the coordination of OH^−^ to the surface of the NPs, which increased the NPs’ electron density and made it possible for the oxidative addition of the O–H bond. In this respect, significant increases in the rate of NaBH_4_ hydrolysis have been accomplished for a variety of metallic NPs [68,69]. However, recent research has revealed that NaOH has a deleterious effect on Pt and Pd NPs [70,71]. These nanoparticles have a very electron-rich structure that allows them to readily help in the oxidative addition of an O–H bond to a surface-bound activated H-bonded adduct [BH_3_H^−^]–H–OH. It is possible that increasing the electron density of the Pt and Pd NP surface is not necessary because it has been hypothesized that the additional hydroxide ion will occupy surface active sites, which will hinder substrate activation [68,72,73]. During the catalyst preparation as shown in FTIR spectra, OH groups are formed due to the executed reduction process in a methanol medium. Thus, the addition of NaOH has shown a detrimental influence on the catalytic performance of Ni_0.3_Co_0.7_@PVDF-co-HFPNF membrane catalyst for the catalytic hydrolysis of NaBH_4_.

#### 3.2.4. Effect of the Temperature in the Hydrolysis Reaction

**Figure 9:**
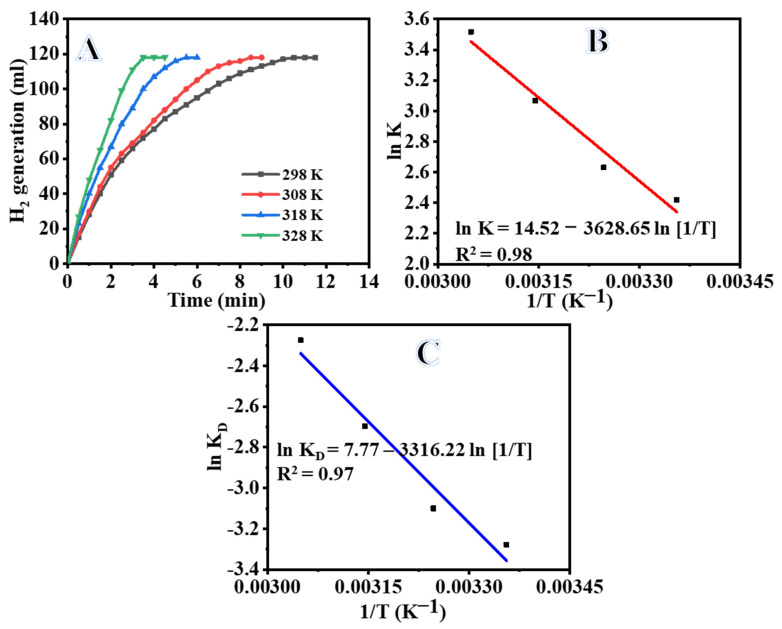
Influence of temperature on the dehydrogenation of SBH (**A**), the logarithmic value of the K for H_2_ production as a function of the inverse of the temperature (**B**), and the logarithmic value of the K_D_ as a function of the inverse of the temperature (**C**). [Amount of catalyst = 100 mg of Ni_0.3_Co_0.7_@ PVDF-co-HFP NFs membrane and [SBH] = 1 mmol].

Peak hydrogen production in the hydrolysis of SBH is strongly influenced by temperature. As all other factors are held constant, more hydrogen is produced when the reaction temperature is increased. Hydrogen production is shown to increase linearly with temperature over a Ni_0.3_Co_0.7_@PVDF-co-HFPNF membrane catalyst, as shown in Figure 9A. The maximum volume of H_2_ generation (118 mL) was achieved in 11.5, 9, 6, and 4.5 min at 298, 308, 318, and 328 K, respectively. By increasing the temperature of the reaction from 25 to 45 °C and using cobalt as a catalyst, Kao et al. [74] showed that the catalytic dehydrogenation of solid hydride is strongly temperature sensitive, which leads to an increase in the rate of the reaction.

#### 3.2.5. Evolution of Thermodynamics of the Hydrolysis Reaction

Arrhenius and Eyring charts (Figure 9B,C) are used to compute the thermodynamics parameters at each temperature (298 to 328 K). In order to perform the linear regression, a plot of the logarithmic rate vs. the inverse of the temperature was drawn, as illustrated in Figure 9B,C. Given this, the slope of the linear plot revealed the *E_a_*, Δ*S*, and Δ*H* values, which were found to be 30.17, 0.065, and 27.57 kJ/mol, respectively. In addition to this, it is stated that the measured Ea is passably lower than what was obtained in the related literature. In Table 1, a comparison of the activation energies of several supported and unsupported catalysts, Co-based catalysts, and Ni-based catalysts is presented.

#### 3.2.6. Reusability Test of Ni_0.3_Co_0.7_@PVDF-co-HFP NF Membranes Catalyst in the Hydrolysis Reaction

**Figure 10:**
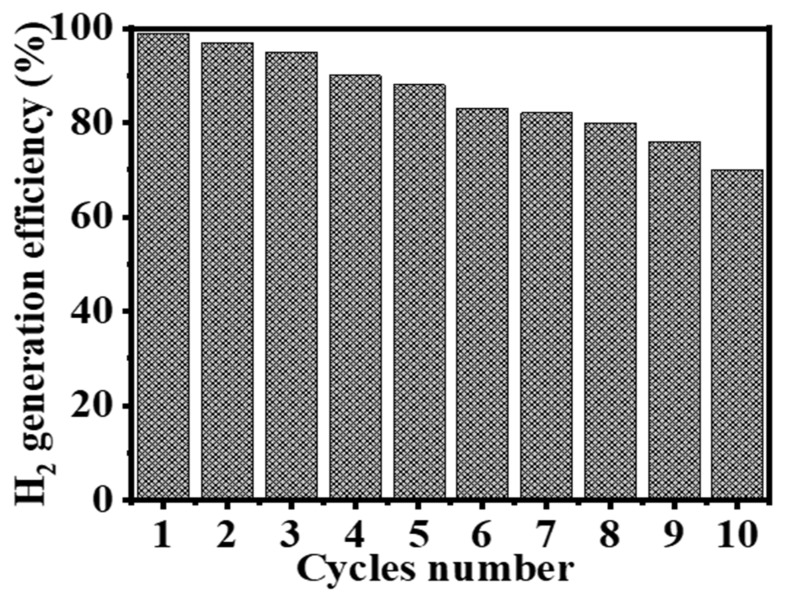
Reusability test of the Ni_0.3_Co_0.7_@ PVDF-co-HFP NF membrane. [Quantity of catalyst = 100 mg, [SBH] = 1 mmol, and T = 298 K].

**Figure 11:**
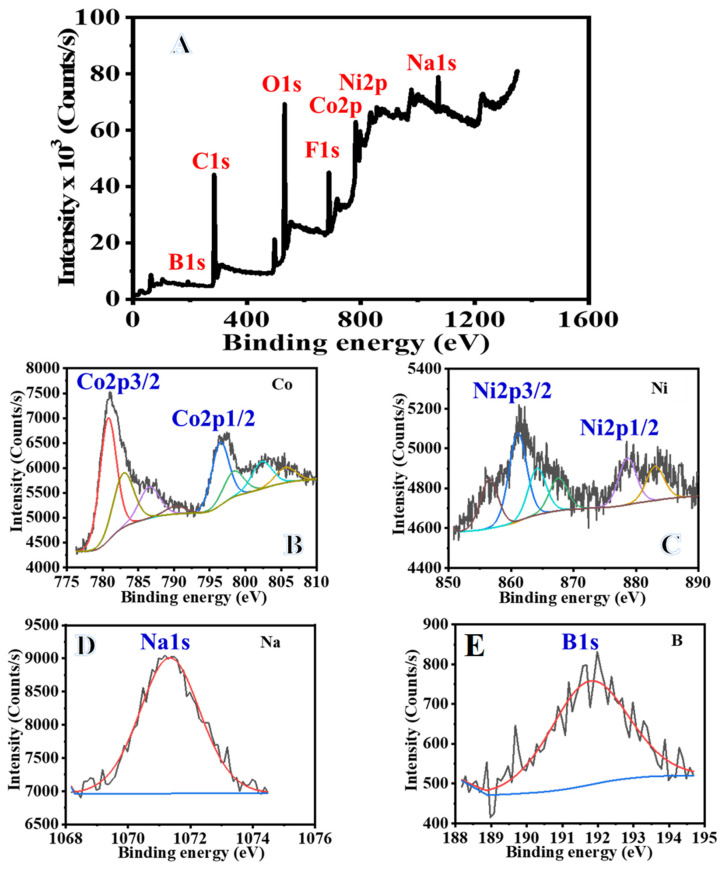
XPS spectra of the Ni_0.3_Co_0.7_@PVDF-co-HFP NF membrane after ten reused cycles: full spectrum (**A**), Ni2p (**B**), Co2p (**C**), Na1s (**D**), and B1s (**E**).

The practical application of catalysts places significant emphasis on the durability of the catalyst. In this work, the hybrid NF mat that was introduced is straightforward to disassemble and recycle. In light of this, the hydrolysis of 1 mmol of SBH at a temperature of 298 K was carried out using a catalyst consisting of 100 mg of Ni_0.3_Co_0.7_@PVDF-co-HFP NF membrane catalyst. Following the completion of the hydrolysis of SBH, an additional 1 mmol of SBH was added to the same solution in preparation for the subsequent cycle. This was undertaken without isolating the Ni_0.3_Co_0.7_@PVDF-co-HFP NF membrane catalyst first. As can be seen in Figure 10, at the end of each run, a complete hydrolysis reaction was obtained, which corresponds to a conversion rate of one hundred percent for SBH after the first cycle. Catalytic activity, on the other hand, gradually decreased as the run numbers increased, reaching 70% of its initial level at cycle 10. It may be possible that reaction products precipitate out onto the surface of the membrane whenever it is reused without being cleaned between cycles. This blocks the metal active sites and slows down the production of H_2_. It has been postulated that an increase in boron products on the membrane surface and an increase in solution viscosity, both of which impede active site accessibility or induce pore blockage, are responsible for the slight decrease in catalytic activity that is observed after the first cycle [87]. This is because both of these factors cause pore blockage. This was confirmed by XPS after six cycles of reuse (Figure 11). In the full-scan XPS spectra of the used sample after six cycles, significant peaks of O 1s, C 1s, and F 1s were identified at 534.1, 286.4, and 688.1 eV, respectively (Figure 11A). The weakest peaks of Ni 2p3/2 and Ni 2p1/2 are indicated at 856 and 874.1 eV, respectively, which are in agreement with earlier findings (Figure 11B). It is important to highlight that Ni 2p peaks are found very frequently due to the structure of the produced sample containing a low concentration of Ni. In the XPS spectra of the used sample, the stated peaks appeared at 780 and 797.2 eV. These peaks are attributed to Co 2p3/2 and Co 2p1/2 peaks, and they contain identical chemical information as observed in Figure 11C. The binding energy associated with B1s is 192 eV (Figure 11D), and the binding energy associated with Na1s is 1071.2 eV (Figure 11E). One of the possible explanations for this phenomenon is that the catalyst is covered in Na ions, which is mostly caused by the presence of byproducts such as NaBO_2_. These results provide new insight into the use of hybrid NF mats for H_2_ production from various hydrogen storage materials and help the design of new advanced catalysts.

The kinetics and thermodynamics of the hydrolysis process based on the influence of the amount of Ni_0.3_Co_0.7_@PVDF-co-HFPNF membrane catalyst, NaBH_4_ concentration, and temperature may be expressed in the following equations:

(2) $$r=-\frac{d\left[SBH\right]}{dt}=k{\left[{\mathrm{Ni}}_{0.3}{\mathrm{Co}}_{0.7}@\mathrm{PVDF}-\mathrm{co}-\mathrm{HFP}\right]}^{1.046}{\left[SBH\right]}^{0.019}$$

(3) $$k=Ae^{\left(\frac{-E_{a}}{RT}\right)}\to lnk=ln14.52-\frac{30,169}{8.314T}$$

(4) $$r=-\frac{d\left[SBH\right]}{dt}=14.52e^{\left(\frac{-3628}{T}\right)}{\left[{\mathrm{Ni}}_{0.3}{\mathrm{Co}}_{0.7}@\mathrm{PVDF}-\mathrm{co}-\mathrm{HFP}\right]}^{1.046}{\left[SBH\right]}^{0.019}$$

The Δ*H* and Δ*S* could be utilized to obtain Δ*G* utilizing Equations (5) and (6).

(5) $$\ln k_{D}=\ln\frac{k_{B}}{h}+\frac{\Delta S}{R}-\frac{\Delta H}{RT}$$

(6) ΔG=ΔH−TΔS

The values of Δ*H* and Δ*S* are calculated to be 27.57 kJ mol^−1^ and 0.065 kJ mol^−1^, respectively, using Equation (6) in Figure 9C. The Δ*G* may be stated in a simple way as follows:

(7) ΔG=27.57−0.065T

## 4. Conclusions

Electrospinning, followed by in situ reduction, proved to be an effective method for the synthesis of NiCo/PVDF-HFP NF catalysts, which were then utilized to successfully generate hydrogen from sodium borohydride. The electrospun bimetallic catalysts exhibited a high level of catalytic activity as a result of the synergistic effects that are caused by the interaction of the metals. When Ni_0.3_Co_0.7_@PVDF-co-HFP was applied, the generation of H_2_ reached the highest and fastest level possible. The rates of hydrogen production were determined to be 11.3, 18.4, 23.6, and 29.5 mL/min for catalysts containing 100, 150, 200, and 250 mg of Ni_0.3_Co_0.7_@ PVDF-co-HFP NF membrane catalyst, respectively. In terms of the concentration of SBH, the hydrolysis reaction follows a zero-order reaction mechanism. It was determined that a comparable activation energy of 30.17 kJ/mol was obtained. Additionally, the polymeric electrospun catalysts are simple to separate, reuse, and refabricate once they have been used.

## Figures and Tables

**Table 1:** Ea of membrane NFs, Ni, and Cu-based catalysts for the dehydrogenation of SBH.

Catalyst	E_a_ (KJ/mol)	Ref.
Ni	42.28	[74]
Ni	71	[14]
Raney Ni	63	[14]
Ni(0)	51.4	[71]
Co@ hydroxyapatite	53	[75]
Co@carbon nanospheres	40.79	[50]
Co@ three-dimensional graphene oxide	55.22	[76]
Co-B@ graphene oxide	26.2	[77]
Ni-Co	38	[78]
Ni-Co-B	62	[79]
Co-B/Cu	43.3	[80]
Co-Ni@ reduced graphene	55.2	[18]
Co-B/Ni	94.5	[81]
Co-B/Cu	43.3	[82]
Co-Ni-B	33.1	[1]
Co-Ni/AC	68.9	[9]
Co-Ni/MWAC	40.7	[83]
Sm-Ni-Co-P/g-Al_2_O_3_	52.1	[84]
Ni–Co–B	62	[79]
Ni-hollow PVDF capsules	49.3	[85]
Ni-PVDF hollow fiber	55.3	[28]
([C6(mpy)2][NiCl4]^2−^	56.4	[86]
PVDF-[C6(mpy)2][NiCl4]^2−^	44.6	[15]
Ni-Co@PVDF-co-HFP	30.17	This study

## Data Availability

The data presented in this study are available from the corresponding authors upon reasonable request.
